# Adult Stem Cells for Cartilage Regeneration

**DOI:** 10.7759/cureus.32280

**Published:** 2022-12-07

**Authors:** Omar M Ismail, Umar N Said, Omar M El-Omar

**Affiliations:** 1 Faculty of Biology, Medicine and Health, Manchester Medical School, The University of Manchester, Manchester, GBR

**Keywords:** chondrogenic cells, collagen degradation, human embryonic stem cells, osteoarthritis, mesenchymal stem cells (mscs)

## Abstract

As cartilage is an avascular, aneural structure, it has very low capabilities of self-repair. Osteoarthritis prevalence is increasing, and there are no clinically approved management techniques that can cure the degradation of cartilage. This report investigates the efficacy of different sources of cells to generate articular cartilage. Autologous chondrocyte implantation has been used to some extent in clinics; however it has not generated efficient, reliable results, and there is no evidence of long-term success. The usage of stem cells is more promising, particularly mesenchymal stem cells (MSCs). Human embryonic stem cells (hESCs) have also been trialed; however, it is important to note that the process of differentiation into chondrocytes is not fully understood, and the cartilage produced can often be of poor quality. MSCs seems to be the way forward, and hESCs will perhaps need further study with the usage of MSC differentiation methodology.

## Introduction and background

Cartilage is considered to be avascular and aneural - meaning that it has no vessels or nerves running through it [1]. This connective tissue is found in joints at the end of all long bones and is referred to as articular cartilage [1]. It consists of only one type of cell, the chondrocyte [1]. The chondrocyte secretes and organizes an extensive extracellular matrix (ECM) [1]. This is the main component of articular cartilage (apart from water) and chondrocytes are embedded within it, but these only form 1-2% of the tissue mass [2].

**Figure 1:**
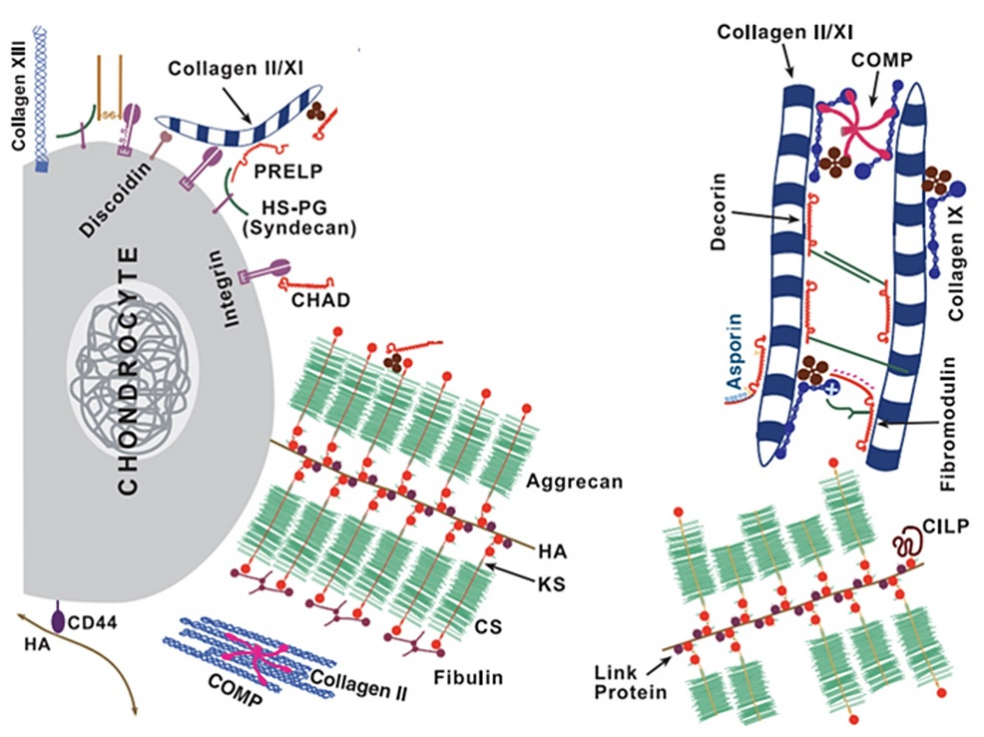
Schematic representation of articular cartilage Schematic representation of articular cartilage. The networks of collagen are evident, as is the aggrecan aggregation with hyaluronan and link protein. Adapted from [1] with permission.

The ECM is mainly made up of collagen fibers embedded in proteoglycan [1, 2]. The collagen (predominantly type II, with lower amounts of types IX and XI) gives tensile strength, whilst the proteoglycans provide the cartilage with elasticity and the ability for the cartilage to resist compression [2]. Of the proteoglycans, the most abundant is aggrecan (or cartilage-specific proteoglycan core protein) [2]. Large numbers of aggrecan bind to hyaluronan through link-protein to form supramolecular aggregates (Figure 1) [2]. Immobile anionic groups are attached to aggrecan which attract mobile cation counterions [2]. This then draws water (a polar molecule) into the cartilage matrix, and this increase in water extends the network of collagen fibers until there is an equilibrium between the tissue swelling pressure and tension of the collagen [2].

Around 75% of articular cartilage is made up of water, and its elasticity and compressive resilience result from both the hydrated fibrillar collagen and the aggrecan proteoglycan [1]. As a result, it is crucial that the collagen network and aggrecan surrounding it in the ECM remain intact and are maintained over time [2]. However, as a result of this specific interlinked structure with a lack of neurovascular structures (which would allow systemic cells to interact with the local environment), articular cartilage has a very limited ability to repair once it is damaged [2]. Cartilage trauma, which can commonly occur in sports, for example, can lead to serious joint injuries and osteoarthritis [2].

## Review

Osteoarthritis

In order to further set the scene for this report, it will be necessary to consider the illness that requires cartilage regeneration.

Epidemiology

Of the different forms of arthritis, osteoarthritis (OA) is the most prevalent [3]. OA predominantly affects the spine, hip, knee, interphalangeal and ankle joints [3]. The prevalence of people in the United Kingdom with OA in at least one knee is over six million people [4]. Approximately a fifth of adults between the ages 50-59 have painful OA in at least one knee, and this prevalence increases to approximately half of adults aged 80+ [4]. OA is less common in the hip joint; at least 650,000 people in the UK suffer from painful OA in at least one hip joint [5]. Those aged over 65 years make up three-quarters of this [5]. However, the most common form of OA is found in the spine [6]. Arthritis Research UK [6] affirms this by stating that "there are almost 8.5 million people with x-ray evidence of OA of the spine in the UK." Interestingly, it is different from OA in other areas of the body in that it is more prevalent in men than women (by a ratio of 3:2) [7]. 

Etiology

The etiology of primary OA is unknown [3]. There are, however, various factors that can predispose OA. Some of these include trauma (OA at joints can be caused by ligament damage [3]), occupation (manual laborers and sportspeople will be very active and cartilage at certain joints will wear thin faster as a result [3]), osteoporosis (OA is less likely in this event [3]), and pre-existing joint damage (such as rheumatoid arthritis, septic arthritis, gout, and avascular necrosis [3]). It should also be noted that there may be a familial or genetic element to OA [3]; however, genes responsible for collagen type II production have not been singled out yet [3]. There are ongoing studies to determine which genes have an effect on the risk of developing OA [8]. OA also has environmental components, namely aging and biomechanical stress [8].

Pathogenesis

In the eighth edition of their textbook Pathologic Basis of Disease (2010) [8], Robbins and Cotran state that: "it is an oversimplification to consider OA an inevitable consequence of cartilage wear and tear." Indeed, although the pathophysiology of OA is not totally understood, it is important to note that chondrocytes are very much involved [8]. There are numerous stages of OA: firstly, chondrocyte injury (which can arise over time due to aging, biochemical and genetic factors) [8] and this affects the equilibrium between production and degradation of cartilage by chondrocytes [3]; secondly, early OA which is characterized by the proliferation of chondrocytes, which then release collagens, inflammatory mediators, proteases, and proteoglycans which all remodel the cartilaginous matrix and can lead to secondary inflammation in the subchondral bone and synovium [8]; finally, late OA which constitutes chronic inflammation (resulting from repetitive injury), leading to a loss of cartilage and changes in structure and shape to the subchondral bone [8].

**Figure 2:**
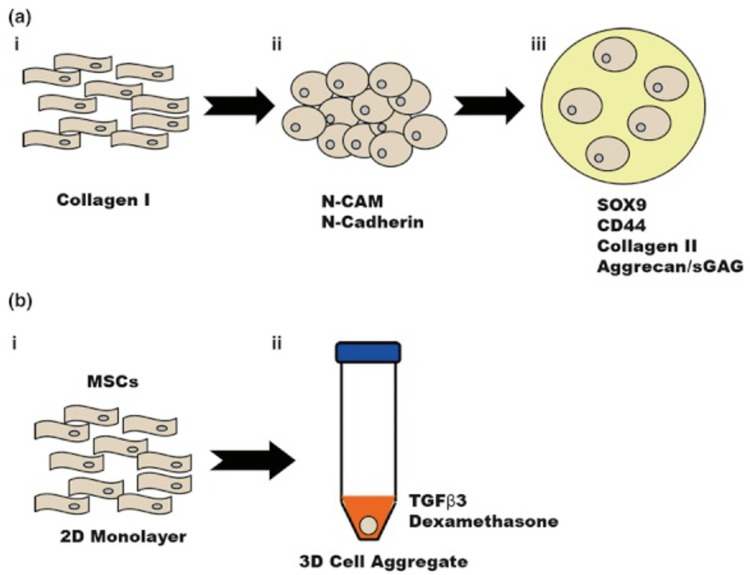
Chondrogenesis of MSCs in vivo and in vitro. (a) *In vivo* chondrogenesis. (a.i) Chondrogenesis begins during embryonic development with MSCs expressing collagen I. (a.ii) MSCs migrate and form mesenchymal condensations which activate molecular signalling cascades from plasma membrane receptors, including N-CAM and N-Cadherin. (a.iii) MSCs then proceed to undergo chondrogenic differentiation, depositing cartilage-ECM molecules (such as CD44, collagen II and aggrecan) under the influence of SOX9. (b) *In vitro* chondrogenesis. (b.i) MSCs are aggregated *ex vivo* in a 2D-monolayer. (b.ii) MSCs are then added to a 3D-cell aggregate and are grown in this medium. Following this, *in vitro* differentiation is then the same as *in vivo*. Adapted from [10] with permission.

In early OA where there is a proliferation of chondrocytes, the water content of the matrix increases whilst there is a decrease in the concentration of proteoglycans (due to the structure of proteoglycan and cartilage falling apart, which allows water to enter the cartilage) [8]. As a result, cracking of the matrix is evident as well as degradation of cartilage and collagen type II molecules [8]. This precedes the shedding of entire full-thickness sections of cartilage as chondrocytes die [8]. Loose cartilage and pieces of the subchondral bone can fall into the joint, resulting in residual bodies (Figure 2) [8]. The subchondral bone plate is now exposed, and thus replaces the old articular surface [8]. The friction generated between both exposed subchondral bone plates results in smoothening of the surfaces [8]. At the same time, there is sclerosis of the cancellous bone underneath the surface [8]. Following this, minute fractures arise through the bone and these spaces give way to the synovial fluid being forced into the subchondral area [8]. This loculated fluid collects and pools which forms fibrous-walled cysts [8]. At the edges of the articular bone, mushroom-shaped osteophytes (bony spurs) arise, and are covered by hyaline cartilage and also fibrocartilage, which eventually undergoes ossification [8]. The synovium becomes slightly congested and fibrotic, and chronic inflammatory cells may also be evident [8].

Clinical Features

OA is considered to be a significant cause of disability, particularly when it affects the hips and knees [3]. It is important to note that early OA is often asymptomatic [3], and flare-ups may be a result of inflammation accompanied by a slight rise in C-reactive protein (CRP) [3]. Symptoms may include joint pain, joint gelling (stiffening and pain after immobility), joint instability, and loss of function [3]. Some signs of OA consist of joint tenderness, crepitus (grating noises) on movement, a limitation in the range of movement, joint instability, joint effusion and variable levels of inflammation, bony swelling, and wasting of the muscles [3].

Current methods of managing osteoarthritis

As OA is becoming an increasingly prevalent disease that has the potential to seriously debilitate some of those affected, there is ongoing research into finding a way to treat OA. In this section, the current guidelines for managing OA will be considered.

Physical Management

In a consultation, the doctor must always consider how to holistically treat the patient by assessing the effect of OA on the patient's mood, occupation, and quality of life [9]. The doctor can then make an individual plan tailored to the needs of the patient with an emphasis on self-management to maximize the effectiveness of positive behavioral changes [9].

There are various core treatments that can be advised for all patients [9]. Firstly, there is weight loss and exercise to strengthen local muscle and improve general aerobic fitness [3, 9]. There is increasing evidence that promotes acupuncture for knee OA [3]; however for now the National Institute for Health and Care Excellence (NICE) does not recommend this treatment for OA [9]. The doctor and patient may also wish to try other complementary medicine treatments which, despite a lack of scientific evidence, may be worth trying as a few patients do respond well to this treatment [3].

Depending on the patient's needs and preferences, other treatments may be offered [9]. Thermotherapy and electrotherapy can act in addition to core treatments to for pain relief [9]. Moreover, bracing/joint supports/insoles can be offered to patients who have biomechanical joint pain or instability symptoms [9]. Walking sticks held on the contralateral side of the affected lower limb can also be helpful [3, 9].

Pharmacological Management

Clinicians should consider offering paracetamol and/or topical non-steroidal anti-inflammatories (NSAIDs) for pain relief as an adjunct to the core treatments [9]. If these are not effective, oral NSAIDs and opioids can also be considered [9]. Finally, intra-articular injections can be considered, and these contain corticosteroids that can also treat joint pain and inflammation in OA [9]. These provide short-term pain relief, but frequent injections should be avoided as this could lead to lasting damage [3, 9]. Unfortunately, there are no drugs that can stop or cure OA [3].

Surgery

Doctors can contemplate referring patients for joint surgery if they suffer from pain, stiffness, and reduced mobility in their joints that affect their quality of life and where non-surgical treatments are less effective [9]. This should be considered the last option as it is an invasive procedure [3, 9]. Knee arthroscopy is not advised in the vast majority of cases [3, 9]. Instead, it is advised to perform a total knee replacement or unicompartmental knee replacement [3]. For the hip, NICE recommends total hip replacement or hip resurfacing [9]. Care should be taken to ensure prostheses are used where the rate of revision is lower than 5% at 10 years [9]. These surgical procedures greatly reduce pain and stiffness and improve mobility and function [9]. However it should be noted that these are not curing treatments for OA, as the prostheses can only last for so long, and any revision will not give the same improvement the initial replacement gave [9]. Thus, with younger patients, the clinician should be frank about the risks and benefits of these procedures unless in extreme circumstances (for example after certain traumas or with end-stage avascular necrosis).

Genicular nerve radiofrequency ablation (GNRFA) has also been used for the symptomatic management of osteoarthritis [10]. In cases where conservative treatment has not been helpful and surgery is either not appropriate (due to co-morbidities for example) or has failed [10]. Studies have shown it can provide short-term benefits for pain in patients [10]. This procedure is performed under local anesthetic or sedation where a cannula is placed close to the genicular nerves and emits a radio wave that cauterizes pain-transmitting nerve fibers [10]. Risks with this procedure are fairly rare but patients should be aware that it can lead to skin burns or vascular injury [11]. 

Autologous Chondrocyte Implantation (ACI)

One of the earliest developing tissue engineering techniques for regenerating articular cartilage is autologous chondrocyte implantation (ACI) [10]. Autologous refers to taking cells from the same person. In ACI, articular cartilage biopsies are removed from the low-weight-bearing areas of the patellofemoral joint (so that the adverse impact of removing cartilage from one area is minimal) [12]. Autologous chondrocytes are taken and undergo ex vivo expansion so that they can be implanted into damaged weight-bearing surfaces, where there are high levels of cartilage degradation [12].

At first, surgical procedures for ACI consisted of suturing a periosteal flap to fix the autologous cells to the graft site [13]; however, later it was found collagen membranes could be used instead of a periosteal flap in order to reduce the time of operation and to reduce possible complications that involve graft hypertrophy [14]. However, after there were problems discovered concerning cell leaking from the site of implantation, further ACI procedures have developed such as Matrix-Assisted Chondrocyte Implantation [15, 16]. This is a faster method that is brought about using a collagen type I/III membrane, which improves the fixation of cells (so leaks are prevented) and improves the distribution of chondrocyte tissue around the site of implantation [15, 16].

ACI (and its other advances and adaptations) has been adopted in clinics with more frequency over time and is the only clinically proven tissue engineering technique concerning regenerating articular cartilage [10]. In spite of this, there are some limitations. Articular cartilage tissue has a low cellularity, and so chondrocytes are expanded ex vivo in order for there to be a sufficient number of cells to treat the degraded areas of cartilage [10]. Mature chondrocytes do not have a high rate of cell proliferation in single-layered culture [10]. Once they are taken out of a 3D extracellular matrix, these cells lose their round shape and dedifferentiate, losing their phenotype (and thus, experience replicate senescence) [10]. As a result, alternatives are needed for larger-scale regeneration as the low rate of cell proliferation, and the tendency for the chondrocytes to dedifferentiate will mean that ACI can only be used for relatively small injuries [10]. Thus, it is not a considered "true" treatment for OA, but rather for young patients with focal cartilage defects. Nonetheless, studies have shown improvements in Knee injury and Osteoarthritis Outcome Scores (KOOS) over a five-year follow-up, with no notable adverse effects [17].

Stem cells

Stem cells are undifferentiated cells that do not undertake any specialized function [18]. However, they have the ability to differentiate into one or more types of mature, specialized cells, such as hepatocytes, skin cells, or chondrocytes [18]. The ability to differentiate into one or more types of mature cells is called "developmental plasticity", and different stem cells have varying degrees of potency [18].

There are two main types of stem cells: adult stem cells and embryonic stem cells [18]. Embryonic stem cells make up the early human embryo, and early in development, these are known as totipotent stem cells; meaning they have the capability to divide into any type of mature and fully differentiated human cell [18]. About four days after fertilization, though, the blastocyst is formed where the outer cells go on to form the placenta and other accessory pregnancy tissues, whereas the inner cell mass is called pluripotent stem cells [18]. The developmental plasticity is reduced, and these cells can develop into any cell type barring the accessory organs of pregnancy [18]. As further development takes place, these cells lose more potency [18].

Adult stem cells are not very numerous and only small numbers can be found in mature organs and tissues [18]. These cells divide mitotically, where one of its daughter cells remains a stem cell yet the other one differentiates into a mature specialized cell [18]. Some adult stem cells are known to be multipotent, meaning that they have the ability to differentiate into at least two different cell lines [18]. For instance, some multipotent bone marrow stem cells (also known as hematopoietic stem cells) can become red blood cells, one of five types of white blood cells, or platelet-producing cells [18]. Unipotent stem cells are only able to differentiate into one type of mature cell, like the cells that differentiate to become sperm cells or egg cells [18].

Cell sources for articular cartilage regeneration

Stem cells have been used in tissue engineering more and more recently, and xenotransplantation has led to scientists being able to grow human ears on the back of mice [18]. It is important to consider which type of stem cell could be used to regenerate articular cartilage, either mesenchymal stem cells or human embryonic stem cells.

Mesenchymal Stem Cells (MSCs)

MSCs were first discovered within the stromal compartment of the bone marrow [10]. At first, they were thought to play a supporting role in hematopoietic stem cell differentiation, but in fact have stem cell characteristics themselves, such as cloning expansion by self-renewal and osteogenic differentiation [10, 19]. MSCs have also been discovered to be multipotent and can differentiate into adipocytes, osteoblasts, and also crucially, chondrocytes [20].

MSCs can be taken from tissues such as bone marrow [21], the umbilical cord [22], skeletal muscle [23], and adipose tissue [10, 24]. MSCs have an advantage over mature chondrocytes in that they can undergo expansion in culture more efficiently and to a greater degree, thus appearing as a more advantageous cell source for autologous cell transplants [25]. MSCs also divide and differentiate in vitro in very much the same way embryonic in vivo chondrogenesis occurs (Figure 2) [10].

Undifferentiated MSCs that express collagen I, hyaluronan, tenascin-C, and fibronectin all condense and form cartilage, shortly followed by bone [26]. SOX9 (the chondrocyte transcription factor) is upregulated and this stimulates the secretion of aggrecan, link protein, collagen types II/IX/XI, and other molecules found in the ECM [27]. The resultant cells are then encased within the ECM, and gain the round shape that chondrocytes possess [27].

Concerning in vitro differentiation, MSCs are expanded in high-density cultures with a pro-chondrogenic medium [20, 28]. 24 hours later, cell signaling amongst the MSCs is sufficiently powerful so that continuous tissue is formed [20, 28]. After day seven, the wet mass of tissue increases when cartilage-specific ECM genes are detected [20, 28].

Centrifugation of MSCs results in spherical tissue [10]. Histological analysis shows that there is an outer layer, 2-3 cells thick, which is made up of undifferentiated, flattened MSCs [10], and beneath this, there are MSCs that have the rounded morphology characteristic of chondrocytes, encased within an ECM [10]. In order to produce a more homogenous tissue with more efficient nutrient and gaseous exchange (in order to make the tissue more suitable for tissue engineering application) there is an alternative that involves a Transwell® insert membrane on which high-density aggregates of MSCs are centrifuged [29].

There is a downside to the use of bone marrow-derived MSCs, which is evident during normal developmental processes; in endochondral ossification during embryogenesis, cartilage can lead to skeletal tissue [10]. Chondrocytes can undergo hypertrophy and down-regulate expression of collagen type II and the transcription factor SOX9, which can lead to a collagen type X matrix [10]. The tissue can have blood vessels developing within it, and chondrocytes undergo apoptosis [10]. Osteoblasts then deposit a bone matrix at the site [10]. Endochondral ossification prevention methods are still under investigation, but once this has been cleared then this will significantly improve autologous cell therapy using MSCs [10]. On the other hand, it is also crucial to note that there are ongoing studies that autologous MSCs can be used to regenerate articular cartilage in humans, and there has been encouraging evidence in favor of this tissue engineering technique [30]. Bone marrow-derived MSCs are the most easily sources and has shown benefit ranging from patients with focal cartilage defects [31] to patients with severe OA [32]

Human Embryonic Stem Cells (hESCs)

hESCs are found in the inner parts of the human blastocyst. hESCs have some advantages over using mature articular chondrocytes and MSCs [10]. hESC-derived chondrogenic cells offer more versatility compared to the autologous tissue engineering techniques, where any set of allogeneic cells produced can be used on a variety of patients, rather than just one, thus being a much more efficient and cost-effective tissue engineering therapy [10]. As these cells are considered pluripotent and can be regulated by various transcription factors, they are a valuable cell source for tissue engineering [10].

However, it should be crucially noted that, unlike MSCs, there is not much knowledge on how hESCs can be used for chondrogenesis [10]. Chondrogenesis and cartilage tissue formation have been demonstrated *in vivo*, but investigations into *in vitro* hESCs spontaneously differentiating (whilst being cultured in a suspension within a medium) show much lower levels of chondrocytes, which could mean that the chemical and environmental factors that arise with *in vitro* methods do not give favorable results [33].

There are a number of methods that can give rise to chondrogenesis from hESCs. The first of which is co-cultures of spontaneously differentiating hESCs with mature chondrogenic cells [10]. It has been demonstrated that having a co-culture of hESCs with chondrocytes (the two cultures are separated by a Transwell® insert) had a positive effect on the chondrogenic differentiation of hESCs, as opposed to using SOX9 and collagen II expression [34]. A similar study showed that when using irradiated chondrocytes, after around two weeks in culture, cells were dissociated with collagenase type II enzyme [35]. hESCs which had partially matured and differentiated was inserted back into the cell aggregate and were supplemented with TGFβ3 and dexamethasone [35]. Once again, increased chondrogenesis was evident compared with control cultures [35]. Yet another advance in this method was demonstrated in a study where pro-chondrogenic cytokines were added to the differentiation media in order to improve the ratio of hESC differentiation to chondrocytes [36]. This reduces the variability of the co-culture primary cells in different batches, which gives a greater opportunity to control the concentrations of growth factors and cytokines, in turn giving more control over the product of the tissue engineering technique [36]. For instance, it is possible to include higher proportions of collagen type II in the ECM, so as to be more accommodating to biomechanical movement [6].

Another methodology consists of differentiating hESCs to MSC-like cell mass, and these are then differentiated to become chondrocytes via the earlier described methods [37]. In one study [38], hESCs were aggregated on a tissue culture that was coated in gelatin with a medium containing FGF-2 and PDGF-AB, in order to enhance cell proliferation, expansion, and differentiation to MSC-like populations. As a result, the cells expressed cell surface antigens very similar to those MSCs, and all were able to differentiate into chondrocytes [38].

Despite all these methodologies, much more research and progress are necessary to see it introduced as a clinical therapeutic treatment [10]. Chondrogenic differentiation, as seen, can often be quite poor compared to using mature chondrocytes or MSCs expanded ex vivo[10]. Another possible tissue engineering technique is the targeted differentiation of hESCs to chondrogenic cells [10]. It has already been shown to produce pancreatic β-cells [39], hepatocytes [40], and cardiomyocytes [10, 41]. The technique involved is very similar to how development occurs in embryogenesis, which involves splitting the process into stages [10].

In this tissue engineering technique [42], there is a starting culture with undifferentiated hESCs (with high developmental plasticity), and a culture system alongside a serum-free base containing nutrients maintained the starting culture [10]. This 2D monolayer is exposed equally to nutrients and growth factors, which then leads to homogenous cell development [10]. BMP-4 is added to direct the mesendoderm cell population to a mesoderm population, and FGF-2 is added to promote cell proliferation [10]. In the second stage, activin is removed and any endogenous activin is inhibited using follistatin [10]. Chondrogenic differentiation is then achieved when BMP-4 is swapped with GDF-5, a cytokine that has been identified during articular cartilage development [43].

Chondrogenic cells assemble into an ECM expressing collagen type II, and crucially not type X which can lead to a mineralized bone matrix [10]. On the other hand, despite the fact that this method produces a more homogenous phenotype, there are no studies that have demonstrated the application of such a method in a tissue engineering setting in clinical trials [10].

## Conclusions

Osteoarthritis is becoming an increasingly prevalent problem, particularly in more developed countries where there is an increasing incidence of elderly populations. The avascular, aneural, and composite structure of the extracellular matrix (and the fact that chondrocytes are encased within the lacunae) makes it very hard for articular cartilage regeneration, as degradation is inevitable over time. As invasive and drastic as joint surgery may be, the prosthesis will too degrade over time. Not many results show long-term efficacy with autologous chondrocyte implantation.

MSCs can be easily isolated from multiple areas of the body and can be used as autologous cell therapy. However, bone marrow-derived MSCs have been shown to potentially express collagen type X and thus lead to bone matrix deposition. Human embryonic stem cell-derived chondrocyte therapy is much more developmental, and quite possibly will not be used in clinics for some time. Targeting hESCs for differentiation to chondrogenic cells seems to be the most promising tissue engineering technique for hESC-derived cartilage based on experimental studies; however MSC-derived cartilage has made much more progress in clinical trials, and so it is very probable that MSC-derived chondrocyte therapy will be in clinical use in the not too distant future. Perhaps, to benefit from hESC benefits (such as no need for tissue to be autologous, and speed of process) more trials can look into how MSC differentiation can lead to successful tissue engineering strategies with hESCs.
